# Zoledronic acid prevents decreases in bone mineral density in patients with prostate cancer undergoing combined androgen blockade

**DOI:** 10.1186/2193-1801-3-586

**Published:** 2014-10-08

**Authors:** Satoshi Nishizawa, Takeshi Inagaki, Akinori Iba, Kazuro Kikkawa, Yoshiki Kodama, Nagahide Matsumura, Yasuo Kohjimoto, Isao Hara

**Affiliations:** Department of Urology, Wakayama Medical University School of Medicine, 811-1 Kimiidera, Wakayama, 641-8509 Japan; Department of Urology, Rinku General Medical Center, Osaka, Japan; Department of Urology, Kinan Hospital, Wakayama, Japan

**Keywords:** Zoledronic acid, Prostate cancer, Combined androgen blockade, Androgen deprivation therapy, Bone mineral density

## Abstract

The aim of this study was to evaluate the effect of zoledronic acid (ZA) on bone mineral density (BMD) in patients with prostate cancer receiving combined androgen blockade (CAB) as a first-line androgen deprivation therapy. Patients receiving CAB for prostate cancer without bone metastasis were candidates for this study. Forty-two patients were randomly assigned to receive either ZA or no treatment. BMD were measured at baseline and at 12 months. Bone-turnover markers, including cross-linked N-telopeptide of type I collagen (NTX), C-telopeptide of type I collagen (ICTP), and bone-specific alkaline phosphatase (BAP), were assessed during study periods. Patients on ZA maintained BMD after a year of treatment. Change in T-score from baseline differed significantly between the two groups (P=0.009). An inverse correlation was demonstrated between baseline and change in T-score in the ZA group. While ZA prevented an increase in ICTP and BAP, the increase in NTX was suppressed only in patients with low baseline T-score. ZA prevented a decrease in BMD in patients undergoing CAB, especially those with lower baseline BMD.

## Introduction

Androgen deprivation therapy (ADT) is a standard option for patients with prostate cancer (PCa) who require systemic therapy. While ADT is effective for PCa, this therapy can induce several side effects (Ahmadi & Daneshmand 2013). For example, long-term ADT can lead to decrease bone mineral density (BMD), a surrogate for fracture risk. Moreover, skeletal fractures are negative predictors of overall survival in patients with PCa (Oefelein et al. 2002). Although the clinical guidelines of the National Comprehensive Cancer Network and the European Association of Urology recommend regular BMD measurement in men with PCa undergoing long-term ADT (NCCN Clinical Practice Guidelines in OncologyTM. Prostate cancer [online]; EAU Guidelines on Prostate Cancer [online]), few patients receiving ADT actually undergo BMD testing (Morgans et al. 2013; Nadler et al. 2013). Several randomized controlled trials demonstrated that zoledronic acid (ZA) increased BMD in men with PCa who were receiving ADT (Smith et al. 2003; Ryan et al. 2006; Michaelson et al. 2007; Israeli et al. 2007; Satoh et al. 2009; Casey et al. 2010; Kapoor et al. 2011; Kachnic et al. 2013; Lang et al. 2013). Since combined androgen blockade (CAB) prolonged overall survival in patients with PCa without bone metastasis in comparison to gonadotropin-releasing hormone agonist (GnRH) monotherapy (Akaza et al. 2009), we have adapted CAB for first-line ADT. CAB was reported to affect bone-turnover marker levels when compared with castration alone (Yamada et al. 2008). However, no studies have investigated the effect of ZA on BMD in patients treated with CAB for ADT. Hence, we evaluated the effect of a single infusion of ZA on BMD in patients with PCa receiving CAB as first-line ADT.

## Patients and methods

### Study participants

This prospective randomized trial was performed in the Department of Urology at Wakayama Medical University between July 2009 and August 2013. This study was approved by the Institutional Review Board of Wakayama Medical University (No. 585). All patients between 60 and 80 years of age who had PCa without bone metastasis and who didn’t receive ADT previously were candidates for this study. Bone metastasis was evaluated by radionuclide bone scan before ADT. All patients had an Eastern Cooperative Oncology Group performance status of 0 to 2. Patients were excluded from study if they had scoliosis, osteosclerosis of the lumbar spine, any other spinal diseases, calcification of abdominal aorta, pulpal or periapical infections, history of other malignancy within 5 years, or more than 1.5 times of the upper limit of serum creatinine, aspartate aminotransferase (AST), or alanine aminotransferase (ALT). Patients were also excluded if they had previously received ADT or bisphosphonates within the preceding 12 months and if they had received estrogens, calcitonin, vitamin D, ipriflavone, raloxifene hydrochloride, or any other drug known to affect the skeleton within 4 weeks of randomization. The 42 patients to meet all criteria were randomly assigned to receive either ZA (ZA-treated group; n = 21) or no treatment (control group; n = 21). At the screening visit, BMD of the posteroanterior lumbar spine (L2-L4) was determined by dual-energy x-ray absorptiometry (DXA), and the T-score was calculated. All patients provided written informed consent.

### Study design

The study was a prospective randomized trial to determine whether a single infusion of ZA could prevent a decrease in BMD in men with PCa without bone metastasis who were receiving CAB. Patients were randomized to receive 4-mg of ZA intravenously on day 1 only or no treatment. Patients were allocated considering the lumbar T-score, age, and performance status. Patients in both groups started treatment with a GnRH agonist (leuprorelin acetate) plus an antiandrogen (bicalutamide), and these drugs were continued throughout the study. A 500-mg calcium supplement and a multivitamin containing 400 to 800 IU of vitamin D were recommended once daily during the study. Patients were evaluated at baseline and at 3, 6, 9, and 12 months after initiation of therapy. Bone-turnover markers, including serum type I collagen cross-linked N-telopeptide (NTX), serum C-telopeptide of type I collagen (ICTP), and serum bone-specific alkaline phosphatase (BAP), were measured at baseline and at 6 and 12 months. BMD was measured at baseline and at 12 months.

### Study end points

The primary end point was the change in lumbar spine (L2–L4) BMD from baseline to 12 months, and the secondary end points were skeletal related events (SRE) and the change in bone-turnover markers from baseline to 6 and 12 months.

### Safety assessment

Serum creatinine and calcium were measured at baseline and monitored every 3 months. The occurrence of adverse events (AEs) was evaluated every 3 months and was recorded using the National Cancer Institute Common Terminology Criteria for Adverse Events version 4.0.

### Statistical analysis

This trial was designed with 80% power and a two-sided *t*-test with α = 0.05 to detect a difference of 3.3% yearly in the lumbar spine BMD between the ZA-treated group and the control group (Smith et al. 2001). Based on these parameters and allowing for a 15% dropout rate, a sample size of 40 men (20 per group) was calculated. The means ± standard deviations were recorded for continuous variables. Differences in variables between two groups with continuous distribution, categorical distribution, and ordinal parameters were assessed using Student’s *t*-test, chi-square test and Mann–Whitney U-test, respectively. Changes in BMD and biochemical markers were analyzed by the paired *t*-test. The correlation among variables was analyzed using Pearson correlation. All analyses were performed at the 5% significance level using StatMate IV (ATMS Co., Ltd., Tokyo, Japan).

## Results

### Patient characteristics

Of the 42 patients in the two groups, two patients treated with ZA didn’t present for follow-up DXA scan at 1 year after infusion of ZA. A total of 40 patients (ZA: n = 19, control: n = 21) were included in the analysis on BMD. All patients were receiving a GnRH agonist and oral bicalutamide at study entry with continuation throughout the 12-month study period. Baseline characteristics including age, strategy of ADT, clinical stage, Gleason score, pretreatment prostate-specific antigen, BMD, and biomarkers of bone-turnover were similar when comparing both groups (Table 1).

**Table 1:** **Patient characteristics (n = 40)**

	Zoledronic acid	Control	***P***value
No. of patients treated	19	21	
Age (years)	72.9 ± 4.8	73.4 ± 5.2	0.75
Strategy of ADT			
First-line therapy	12	16	0.37
Salvage therapy	7	5	
T-stage			
T1c	1	0	0.19
T2	5	2	
T3	12	19	
T4	1	0	
N-stage			
N0	15	18	0.59
N1	4	3	
Gleason score			
≤6	2	3	0.25
7	5	9	
≥8	12	9	
Pretreatment PSA (ng/ml)	76 ± 159	37 ± 72	0.33
BMD (g/cm^2^)	1.235 ± 0.21	1.182 ± 0.22	0.45
T-score	0.55 ± 2.04	0.05 ± 1.91	0.46
Biomarker of bone turnover			
Serum NTX (nmolBCE/L)	15.27 ± 4.86	16.13 ± 5.30	0.62
Serum ICTP (ng/ml)	4.02 ± 1.71	4.40 ± 1.65	0.48
Serum BAP (U/L)	11.57 ± 2.85	13.35 ± 4.29	0.13

ADT, androgen deprivation therapy; PSA, prostate-specific antigen; BMD, bone mineral density; NTX, cross-linked N-telopeptide of type I collagen; ICTP, C-telopeptide of type I collagen; BAP, bone-specific alkaline phosphatse.

### BMD

**Figure 1:**
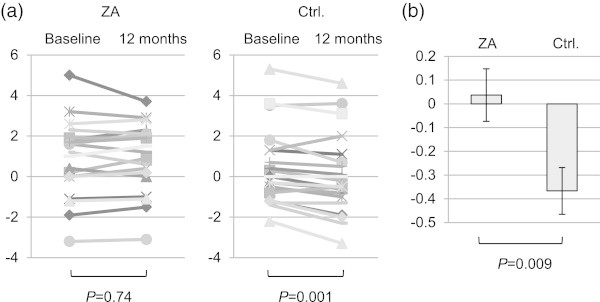
**Change in T-score in the ZA-treated and control groups. (a)** T-score at baseline and 12 months in each group; **(b)** Mean ± SE change in T-score from baseline in the two groups. SE, standard error of the mean; ZA, zoledronic acid; Ctrl, control.

**Figure 2:**
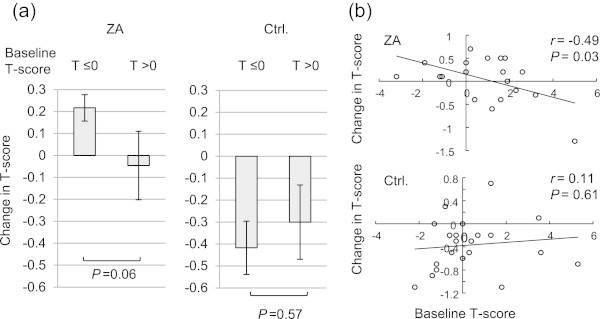
**Change in T-score, as stratified by baseline T-score. (a)** Mean ± SE, as stratified by baseline T-score (T-score ≤0 vs. >0) in each group; **(b)** Correlation between change in T-score and baseline T-score. SE, standard error of the mean; ZA, zoledronic acid; Ctrl, control.

At 1 year after treatment, patients on ZA maintained their T-score (*P* = 0.74), while control patients experienced a significant decrease in T-score (−3.9%, *P* = 0.001). Change in T-score from baseline differed significantly between the two groups (*P* = 0.009) (Figure 1). We performed analysis as stratified by baseline T-score, since the decrease in BMD was considered to contribute to the risk of SRE, especially among patients with lower BMD at baseline. In ZA-treated patients, the lower baseline T-score group (T-score ≤0; n = 6) tended to have an increase in T-score when compared with the normal baseline T-score group (T-score >0; n = 13) (*P* = 0.06), while no difference was revealed in control patients when comparing the lower and normal baseline T-score group (*P* = 0.57) (Figure 2a). An inverse correlation was demonstrated between baseline and change in T-score in ZA-treated patients (*r* = −0.49, *P* = 0.03). By contrast, these variables didn’t correlate with one another in the control patients (*r* = 0.11, *P* = 0.61) (Figure 2b).

### Bone-turnover markers

**Figure 3:**
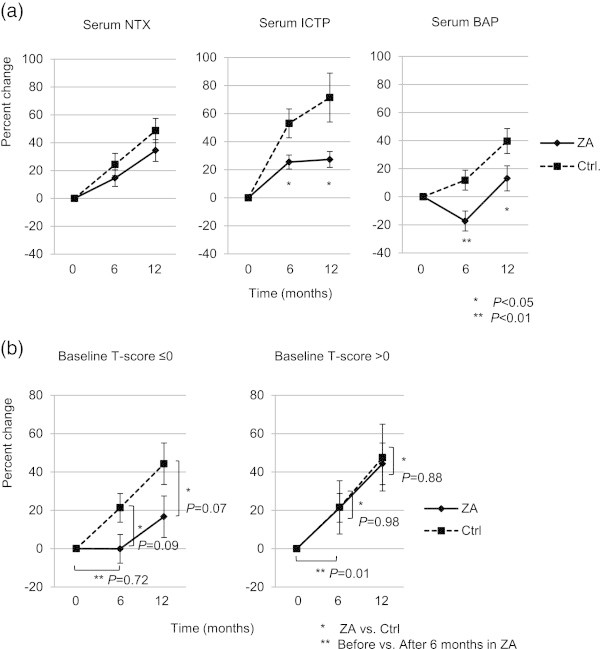
**Comparison of bone-turnover marker levels (mean ± SE). (a)** Serum NTX, ICTP, and BAP were assessed in the ZA-treated and control groups at baseline and at 6 and 12 months; **(b)** Comparison of serum NTX levels, as stratified by baseline T-score (≤0 vs. >0). SE, standard error of the mean; NTX, cross-linked N-telopeptide of type I collagen; ICTP, C-telopeptide of type I collagen; BAP, bone-specific alkaline phosphatase; ZA, zoledronic acid; Ctrl, control.

The change in Serum ICTP and serum BAP at 6 and 12 months were higher in the control group than in the ZA-treated group. However, there was no difference in serum NTX at 6 and 12 months when comparing the two groups (Figure 3a). When stratified according to baseline T-score, in the lower T-score group (T-score ≤0; n = 6) ZA tended to prevent increase in serum NTX compared with control, but this difference didn’t reach the level of statistical significance (*P* = 0.09 and 0.07 at 6 and 12 months, respectively). In the normal T-score group (T-score >0, n = 13), there was no difference in serum NTX when comparing ZA-treated patients and control (*P* = 0.98 and 0.88 at 6 and 12 months, respectively) (Figure 3b). ZA treatment resulted in inhibition of a significant increase in serum NTX at 6 months in the lower baseline T-score group when compared with the normal baseline T-score group (*P* = 0.72 and 0.01 in the lower and normal T-score group, respectively) (Figure 3b).

### Adverse events

There were no AEs higher than grade 3. Neither renal failure nor osteonecrosis of the jaw was reported in ZA-treated patients.

## Discussion

The present study demonstrated that a single infusion of ZA at the time of initiation of CAB for ADT resulted in maintenance of BMD of the lumbar spine in men with PCa without bone metastasis. Although previous several studies showed the positive effect of ZA on BMD among patients treated with ADT (Smith et al. 2003; Ryan et al. 2006; Michaelson et al. 2007; Israeli et al. 2007; Satoh et al. 2009; Casey et al. 2010; Kapoor et al. 2011; Kachnic et al. 2013; Lang et al. 2013), none of the studies investigated men receiving CAB. Non-steroidal antiandrogen monotherapy maintains BMD (Sieber et al. 2004) via maintenance of circulating testosterone levels, elevation of estradiol levels (Smith et al. 2004), and enhancement of osteoblast androgen receptors that act as estrogen receptor modulators (Draper 2003). Moreover, CAB modulates bone metabolism via a decrease in bone-turnover markers when compared with castration alone (Yamada et al. 2008).

The optimal interval and timing of ZA administration is not clear. Other studies used ZA every 3 months (Smith et al. 2003; Ryan et al. 2006; Israeli et al. 2007; Casey et al. 2010; Kapoor et al. 2011). Meanwhile, a single infusion of ZA at the time of initiation of ADT has also been shown to prevent BMD decrease at 1-year after ADT initiation (Michaelson et al. 2007; Satoh et al. 2009), and we confirmed this result in the present study. Rodrigues et al. investigated the optimal schedule of ZA administration in men with PCa undergoing ADT and showed no differences in the change in BMD among four 1-, 2-, 3-, or 6-month ZA intervals (Rodrigues et al. 2010). Hence, only infrequent ZA may be needed for maintenance of BMD in men receiving ADT, which might also extend the benefits of a decreased incidence of severe AEs. Several studies of the timing of initiation of ZA have suggested that early administration of ZA in conjunction with ADT initiation could improve BMD status (Casey et al. 2010; Lang et al. 2013) and that the risk of bone loss is highest during the first year of ADT (Morote et al. 2006). However, delayed ZA treatment also provided some increase in BMD (Bhoopalam et al. 2009).

Several bone-turnover markers have been used to monitor bone metabolism. Since serum levels of bone markers reflect bone resorption and are comparable or superior to conventional urinary markers that have higher biologic variability (Woitge et al. 1999), we used serum bone resorption markers in this study. ICTP was shown to represent pathological bone resorption via rapid breakdown of type I collagen and has been confirmed as an independent predictor of fracture (Meier et al. 2005) and mortality among PCa patients (Jung et al. 2011). However, ICTP didn’t reflect changes in BMD during anti-resorptive therapy (Garnero et al. 1994). In contrast, the skeletal response to alendronate is more apparent when evaluated with another resorptive marker, NTX, suggesting NTX might be a better surrogate therapeutic marker for osteoporosis (Garnero et al. 1994). In the current study, we showed that ZA prevented the increase in ICTP during ADT when compared with the control, which indicates that ZA inhibited pathological bone-turnover. We couldn’t demonstrate the effect of ZA on NTX. However, when restricted to patients with low baseline T-score, we confirmed that ZA tended to prevent an increase in the therapeutic marker, NTX, and actually induced improvement in BMD as a therapeutic effect. Furthermore, in the ZA group, there was continuous inverse correlation between baseline T-score and change in T-score (Figure 2b, *r* = −0.49, *P* = 0.03), suggesting patients with lower baseline T-score retain great benefits from ZA treatment. Israeli et al. also reported patients with low baseline T-score experienced greater skeletal response to ZA than those with normal T-score. (Israeli et al. 2007). However, they only compared between two arbitrary groups (baseline T-score ≤ −1 vs. > − 1), and in view of this, we appropriately reaffirmed the correlation between baseline T-score and BMD change in response to ZA treatment among men receiving ADT. Since patients with low baseline T-score include those who are at risk of fracture, ZA provides great benefit especially in those with low BMD.

Skeletal strength depends on other factors in addition to BMD, and some studies didn’t find significant correlation between BMD and bone-turnover markers. The rate of bone remodeling is a significant factor for bone strength, and bone-turnover markers are independent predictors of fracture (Meier et al. 2005). We should recognize the clinical role of BMD and each bone-turnover marker as above for appropriate bone management.

This study had some limitations. First, the sample size was small and the study period was only 1 year. Second, we studied only one dosing regimen, and BMD was measured only in the lumbar spine. Additional analysis is needed to evaluate the long-term effect of ZA in men receiving ADT.

## Conclusion

ZA administration was safe and well tolerated. A single infusion of ZA prevented a decrease in BMD at 1-year in men without bone metastasis who were receiving CAB. The effect was even great in patients with low baseline BMD.
